# Visual behavior modelling for robotic theory of mind

**DOI:** 10.1038/s41598-020-77918-x

**Published:** 2021-01-11

**Authors:** Boyuan Chen, Carl Vondrick, Hod Lipson

**Affiliations:** 1 Computer Science, Columbia University, Mudd 535, 500 W 120 St, New York, NY 10027 USA; 2 611 CEPSR, 530W 120 St, New York, NY 10027 USA; 3 Mechanical Engineering, Columbia University, Mudd 535E, 500 W 120 St, New York, NY 10027 USA; 4 Data Science, Columbia University, New York, NY 10027 USA

**Keywords:** Mechanical engineering, Psychology

## Abstract

Behavior modeling is an essential cognitive ability that underlies many aspects of human and animal social behavior (Watson in Psychol Rev 20:158, 1913), and an ability we would like to endow robots. Most studies of machine behavior modelling, however, rely on symbolic or selected parametric sensory inputs and built-in knowledge relevant to a given task. Here, we propose that an observer can model the behavior of an actor through visual processing alone, without any prior symbolic information and assumptions about relevant inputs. To test this hypothesis, we designed a non-verbal non-symbolic robotic experiment in which an observer must visualize future plans of an actor robot, based only on an image depicting the initial scene of the actor robot. We found that an AI-observer is able to visualize the future plans of the actor with 98.5% success across four different activities, even when the activity is not known a-priori. We hypothesize that such visual behavior modeling is an essential cognitive ability that will allow machines to understand and coordinate with surrounding agents, while sidestepping the notorious symbol grounding problem. Through a false-belief test, we suggest that this approach may be a precursor to Theory of Mind, one of the distinguishing hallmarks of primate social cognition.

## Motivation: (what is ToM, the challenge, and our hypothesis)

At about age three, a human child recognizes that other humans may have a differing world view than herself^2^. She will learn that a toy can be hidden from a caretaker who is not present in the room, or that other people may not share the same desires and plans as she does. The ability to recognize that different agents have different mental states, goals, and plans is often referred to as “Theory of Mind” (ToM).

In children, the capacity for ToM can lead to playful activities such as “hide and seek”, as well as more sophisticated manipulations such as lying^3^. More broadly, ToM is recognized as a key distinguishing hallmark of human and primate cognition, and a factor that is essential for complex and adaptive social interactions such as cooperation, competition, empathy and deception.

The origins of ToM are difficult to ascertain, because cognitive processes leave no fossil record. In this work, we experimentally look for evidence that ToM would have been preceded by a much simpler precursor which we call “visual behavior modeling”.

Researchers typically refer to the two agents engaged in Behavior Modeling or ToM as “actor” and “observer.” The actor behaves in some way based on its own perception of the world. The observer watches the actor and forms an understanding of the mental state held by the actor and/or the resulting actions that the actor intends to take.

In the simplest case, the actor behaves deterministically, and the observer has full knowledge of the world external to the actor. In more complex cases, observers can have partial knowledge, and agents can be both actors and observers at the same time, simultaneously modeling each other, leading to infinite regress of nested co-adapting models. Here, we study only the simplest case, in both deterministic and stochastic conditions.

Theory of Behavior and ToM experiments are notoriously difficult to carry out because it is very challenging for a researcher to query the state of mind of the observer, in order to determine if the observer truly understands actor’s mental state and plans. In older children and adults, the state of mind of the observer can be queried directly by formulating a verbal question about the actor, such as “Tell me where Sally will look for the ball?” as in the classic “Sally and Anne” test^4–6^.

In young children and primates, as well as in robots, the state of mind of the observer can only be indirectly inferred by inducing an action that reveals the observer’s understanding of the state of mind of the actor. For example, a researcher might ask the child to point at the box where Sally will look for the ball.

A key challenge is that in order to be able to answer a researcher’s question or to follow the researcher’s instructions, the observer must already possess an advanced cognitive ability. For example, the observer must at least be able to understand symbolic questions, make decisions, and formulate responses. The ability to follow instructions, in itself, involves a fairly advanced degree of mental reasoning. This challenge makes it difficult to ascertain the degree to which ToM exists in simple life forms and in robots.

Meltzoff^7^ noted this challenge and proposed the need for non-verbal tests to assess theory of mind. For example, one non-verbal assessment of ToM involves testing whether a child will mobilize to assist another person that appears to be struggling to open a door. Even though these tests are carried out without verbal communication, it is difficult and sometimes impossible for a researcher to conclude with absolute certainty whether the observers performed explicit reasoning while addressing the challenge, or acted instinctively out of some built-in conditional reflex.

Experiments in ToM of robots and AI agents suffer a similar challenge. In a recent set of impressive studies, such as Rabinowitz et al.^8^ agents were able to successfully model the behavior of other agents on a grid world. Baker et al*.*^9^ also demonstrated that complex tool manipulation and coordination strategies can emerge by having adversarial agents play hide and seek in a self-play manner. There are many other examples of AI agents that learn to play successfully against other agents (human and machine) in various video games^10^, frequently outperforming their competitors with impressive gaps. However, in those studies, the players’ inputs involve engineered features and discrete outputs, in the form of grounded symbols, objects, and actions, such as coordinates of agents and obstacles, and discrete actions such as move forward, backward, left or right. This raises the next fundamental question which is where these discrete relevant inputs and actions choices came from, and how were they grounded in the first place.

In some past studies^11^, the observers’ inputs included private internal actor states such as motor commands, that would normally be hidden from an external observer, and therefore could not be used legitimately in a ToM experiment, We focus here on observers that only have access to raw data captured externally using remote sensors, such as cameras and microphones.

Many AI agents use only visual inputs, but most do not explicitly model the behavior of the actor. Instead, they select from a finite repertoire of possible actions that will move them closer towards a goal. For example, Mnih *et al*^10^ showed an impressive example of an agent that learns to play Atari games using only visual inputs, by producing discrete actions that maximize its score. Our own recent work^12^ also shows that having agents play hide and seek with only first-person visual observation can lead to the recognition of the visibility of other agents and self-visibility to emerge, but the action space of the observer agent is still discrete. There is no direct evidence that either system models the plans of the other system. The question of visual behavior modeling is also interesting from an evolutionary point of view. There is no doubt the ToM that involves explicit modeling of actor plans confers an evolutionary advantage to the observer, and may have therefore evolved directly. Foreseeing the plans of an actor can help with many activities beyond competition, such as imitation learning, or cooperation. However, here we explore a precursor question: Can machines intelligently model behavior of other agents without access to explicit symbols, objects and actions?

Being able to model an actor without any explicit symbolic processing could shed light on the evolutionary origin of ToM, as well as offer an alternative path to train machines without the need for feature engineering, symbolic mechanisms, and sidestepping the inductive bias involved in deciding which symbols, objects, and actions need to be built into the system.

**Figure 1:**
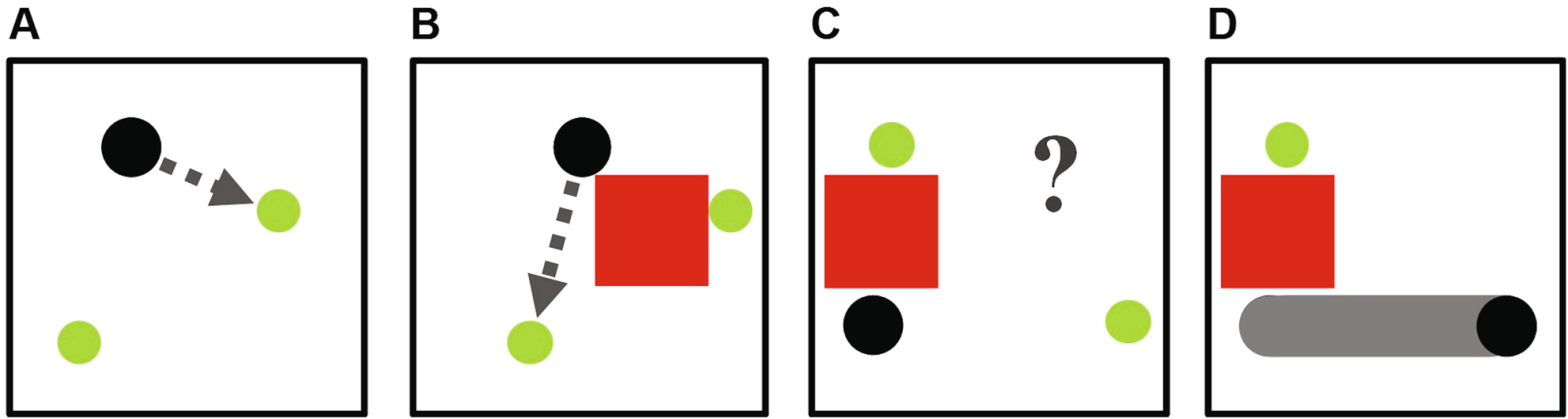
Visual theory of behavior. An actor robot (black circle) is programmed to move towards the nearest food (green circle) that it can see, and consume it. Sometimes (**A**), the nearest green circle is directly visible to the actor, but sometimes (**B**) the nearest green circle is occluded by an obstacle. When occluded, the actor will move towards the closest visible circle, if any. After watching the actor act in various situations, an observer-AI learns to envision what the actor robot will do in a new, unseen situation (**C**). The observer’s prediction is delivered as a visualization of the actor robot’s “trajectory smear” (**D**). This entire reasoning process is done visually, sidestepping the need for symbols, logic, or semantic reasoning.

To test the hypothesis that longer term outcome of behavior can be predicted visually, we focus here on a more abstract notion termed “Theory of Behavior” (ToB)^1^. As shown in Fig. 1, we ask whether an observer can predict the conditional (and possibly stochastic) behavior of an actor based on visual observation of the actor alone, without access to the actor’s internal states, prior knowledge about the behavior. We do not ask whether the observer can ascertain the explicit goal or objective of the actor. We avoid this question for two reasons: First, the explicit goal, if any, of the actor is a hidden variable that may or may not exist, and is subject to interpretation. Second, to process an explicit goal presumes preadapted ability to reason symbolically. In contrast, we hypothesize that the actor can develop the ability to visualize goal-oriented behavior even without having any internal notion of goals at all, and without using symbolic processing ability at all. If true, this suggests a visual precursor to theory of mind.

Our goal is to see if the observer can predict the long-term outcome of the actor’s behavior, rather than simply the next frame in a video sequence, as do many frame-to-frame predictors^11,13–21^. Prediction of next set of frames in a video sequence is both more difficult and less important to a robot that predicting the longer-term outcome of an actor’s behavior. For example, a colleague that watches you rise out of your seat with an empty cup of coffee and head towards the coffee machine, cannot predict your exact movements frame by frame, but can likely predict that you will soon be back sitting at your desk with a refilled cup of coffee. We argue that this precise ability – to envision the outcome of a behavior—is the basis of TOM, not the frame-by-frame prediction that is difficult, yet seems to dominate prior work in visual prediction (probably because there is plenty of data for it).

Visual ToB is thus an end-to-end approach, and therefore it sidesteps many thorny challenges with traditional symbolic reasoning, such as symbol grounding, reasoning with uncertainty, predicate resolution and inference of causality.

## Related work: (the connection between our work with others)

### Theory of mind

There are various definitions of Theory of Mind, as well as various explanations of how it applies to the processes that a human brain performs^22–25^. Most of these can be classified under the so-called Modular Theory, Theory-Theory, Simulation Theory, or some intersections of these three theories.

According to the Modular Theory postulates, there are neural structures specifically designated for Theory of Mind. Two representative models attempting to present a coherent development explanation of this argument are Leslie’s model^26^ and Baron-Cohen’s model^27^. These models differ with respect to the division of perceptual tasks^28^. On the other hand, supporters of Theory-Theory argue that modelling other agents needs to be based on folk psychology^29^ pertaining to how minds of others operate. Finally, Simulation Theory proposes that inferring and predicting the mental state of others involves attempting to see the problem from other’s perspective, i.e., adopting the “if I were you” position^24,25^. These approaches are too high level and human-centered to be directly applicable to robotics.

### Machine theory of mind

Recently, there is an emerging body of work studying the behavior of machines to better understand machine behavior so as to better control it in the future^30^. Researchers have tried to build Theory of Mind that would apply to robots or machines, as this would in their view allow robots to better emulate the ability to grasp a perspective adopted by others. Robot Theory of Mind is also posited to lead to a better understanding of the meta-cognition mechanism and build machines that could exhibit human like behaviors^31^.

In Human Robot Interaction, Scassellati^32^ implemented functions such as face finding and face recognition for different animate and inanimate entities, while attributing them visual attention inherited from humans^33,34^. Systems that try to detect and infer the intentions of humans have also been developed^33,34^. Understanding the behavior of other agents by building and evolving self-models^35–39^ is also a key step in the development of Theory of Mind pertaining specifically to robots.

Authors of recent studies^8,40–43^have also attempted to build a mind network directly for the observer robot, however all with symbolic reasoning processes. We have previously^40^ conducted both simulations and real-world experiments involving a real robot that reverse-engineers the actor’s policy with neural networks based on collected trajectories. However, the trajectories of the actor robot were given to the observer network directly and the definition of the final goal of the actor robot and the dynamics of the scene were not learned.

Rabinowitz^8^ presented a similar problem as a few-shot learning frameworks where the next step action was predicted. Nevertheless, the authors adopted discrete action spaces and experimented on a small (11 × 11) grid world setting. These constraints do not apply to real robotics settings and must be overcome in order to have real life applications.

Moreover, instead of predicting the next step action or discrete goals^8,40–42^, we propose learning a relatively long-term final future sequences of actions and the internal causality structures between the future actions and the final goal of the actor robot. Our method does not need any symbolic reasoning. Moreover, we do not assume that the observer has any prior knowledge about the scene and its dynamics, including the existence of the target objects.

Another branch of the research focuses on modelling multi-agent systems^44–46^. In this work, we only study the cases where we have two agents while one of the agents has Theory of Mind and leave the multi-agent scenario as future work.

### Modelling other agents

Several studies have focused on modelling other agents. For example, Imitation Learning or Behavioral Cloning^47^ is an attempt to mimic the policy of another human or agent. On the other hand, Inverse Reinforcement Learning^48,49^ aims to recover reward functions. Modeling the impact of other agents have also been shown to be useful to stabilize the training process for multi-agent reinforcement learning^50^. Different approaches have also been adopted to build models capable of discerning the behaviors of other agents based on specific interests pertaining to different properties, such as reconstructing the policy or classifying strategy types. We refer the readers to these survey papers^51,52^ for more details.

### Predictive vision

There is a substantial body of work on predictive vision^13–21^. The strategy presented in this paper is also inspired by the successful applications of these approaches. Some research groups are also attempting to utilize this idea on robot motion planning^11^. In these approaches, possible future scene representations are predicted in a recurrent manner, i.e., after the next possible frame is predicted, it is fed into the input loop to predict subsequent frames. As we noted earlier, for the Robot Theory of Mind to have practical applications, it must be capable of predicting both possible future actions and goals of the actor robots over a relatively long-term horizon. However, focusing on the predictions of next frames and accumulating the information that can be inferred from these frames will lead to cumulative errors.

## Experimental setup and results: (how we test our hypothesis)

**Figure 2:**
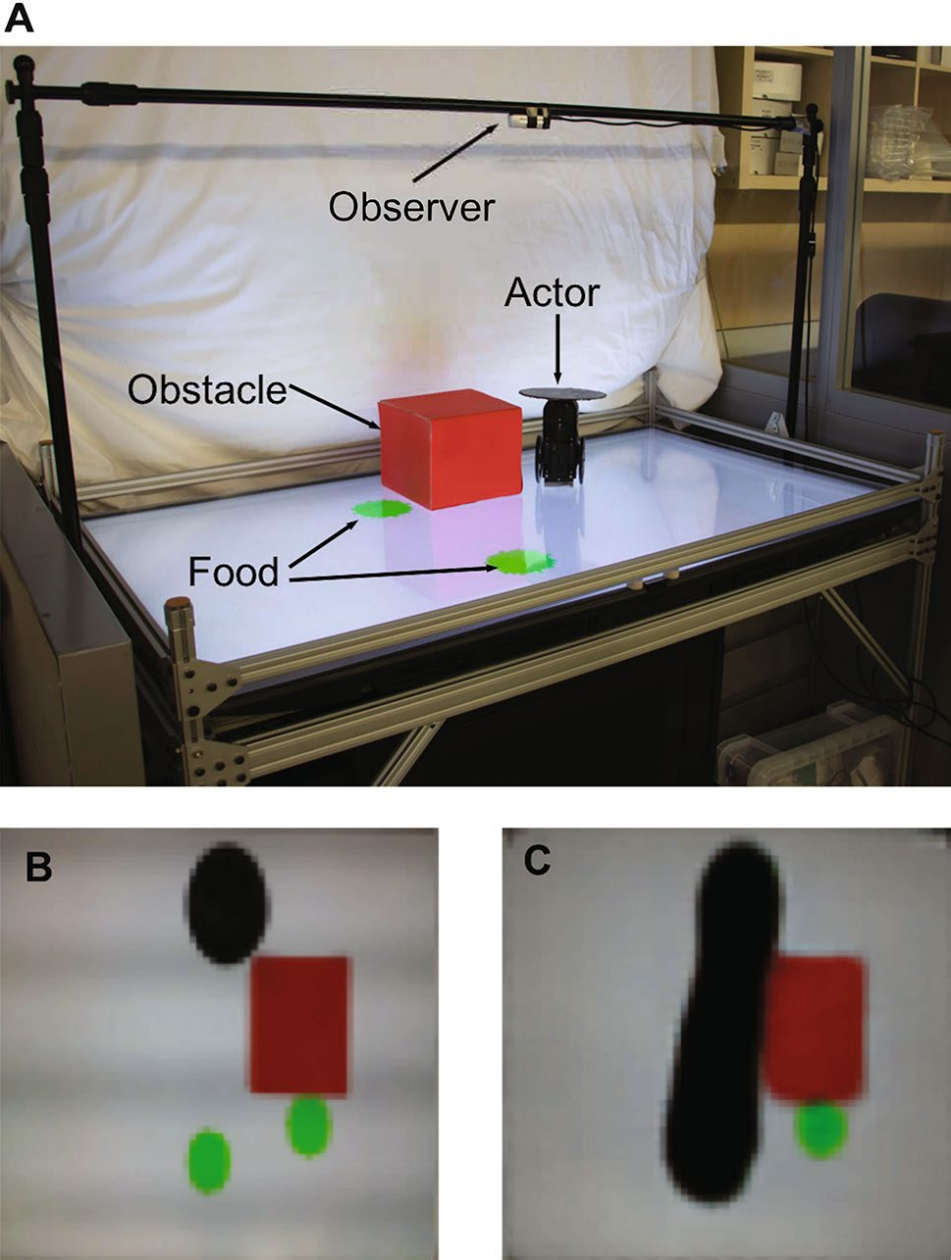
Experimental setup (**A**) Actor robot in playpen, showing observer, green food, and red obstacle. (**B**) A sample input image as seen by the observer, and (**C**) sample output image produced by the observer, which includes a prediction of the motion path of the actor.

In our experiment, we created a robotics system (Fig. 2) comprising a physical robot actor, and an observer agent that watches the actor from above. In order to evaluate the effectiveness of our proposed method, we followed the guidelines proposed by Shevlin and Halina^53^ to design our evaluations from both a Machine Learning perspective and a Psychology perspective. During our experiments, we aimed to answer the following two questions: First, can an observer agent visually predict the future plans of the actor robot, without symbolic reasoning. Second, we are interested in understanding whether the observer agent gains some equivalent to perspective taking abilities.

To test whether the observer can model the behavior of the actor robot from purely visual input, we first pre-programmed (hard-coded) four types of behavior into the actor robot. Without being provided any symbolic information regarding which category of the current actor’s behavior is, the observer machine is asked to generate its envisioned image of what the future scene will look like, including the trajectory and the consequences caused by the actor robot’s behavior. The result is outputted by the observer as a predicted image frame.

The four types of pre-programmed behavior of the actor robot were: (a) Straight line behavior: the actor robot goes to the green circle in a straight line; (b) Elbow behavior: the actor robot goes to the green circle by first navigating towards an intermediate non-colinear point that is located midway and then moves to the green circle in a straight line form there; (c) Zig-zag behavior: the actor robot goes to the circle by navigating through two intermediated non-collinear control points positioned symmetrically on both sides of the straight line path to the goal; and (d) Single-food with obstacle behavior: this behavior is a more complex version of behavior (a) due to occlusion caused by an extra obstacle. In behavior (d), the actor robot goes to the green circle in a straight line if and only if the green circle is visible to it. However, if the green circle is not visible to the actor robot due to the occlusion, the actor robot will not move. When determining visibility, we use a line of sight as the line joining the center of the actor with the center of each food circle, and test computationally whether that line intersects with any obstacle. The actor does not require any physical sensor in this experiment.

**Figure 3:**
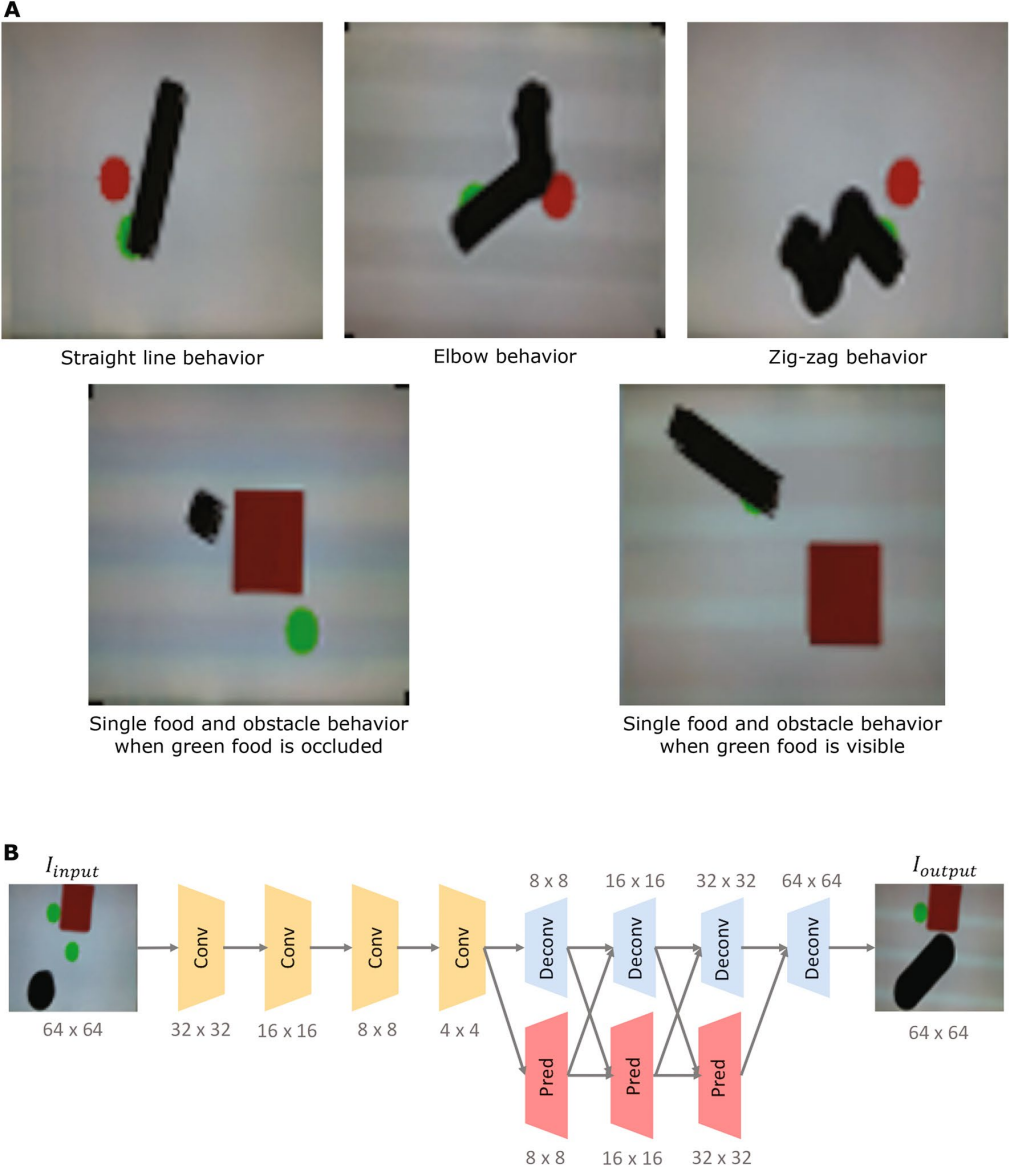
Visualization of the behaviors of the actor robots and Observer network architecture (**A**) We pre-programmed four types of behaviors for the actor robot. The images shown here are produced by integrating a sequence of frames from a video captured by the top-down camera. The robot path is shown in black, the rectangular obstacle (if any) in red and the goal circles in green or red. (**B**) The image prediction network is composed of several layers of convolutional units and deconvolutional units. At the deconvolutional stage, we utilize multi-scale prediction to maintain high resolution at the output image. Numbers indicate the dimension of output feature map after each module.

We show samples of these four behaviors in Fig. 3A, using actual camera shots of the smeared-motion of the robot as seen from the point of view of the observer camera. We use the term “smeared motion” to denote an integration of multiple video frames into a single image by recording the minimum intensity of each pixel across its time history. Thus, a dark robot moving along a trajectory will leave a dark path in its wake.

While some of the four actor’s policies described above involve nested logical clauses, we show that the observer can envision the outcome of those actions without using any explicit logic reasoning, or any explicit notion of objects, obstacles, actions and goals.

The observer AI can only see the world external to the actor using an overhead camera that captures the actor, the green circles and the red obstacle (if any). Importantly, the only information available to the observer are raw camera input images. No labeling, segmentation, motor commands, trajectory coordinates or other derived information is provided to the observer other than the raw camera image itself. The observer is not told, for example, that green pixels are associated with goals, or that colors and shapes even matter. The key question is whether the observer will learn that whether and where the actor moves depends on what the actor can see from the point of view of the actor, not what the observer itself sees.

Importantly, the observer does not output a symbolic answer, such as which green circle will be consumed, which direction the actor will move, or whether or not the actor will move. Instead, the observer envisions the outcome of the actor’s actions holistically by producing a single image of the resulting world, without any explicit knowledge of circles, obstacles, actions or trajectories and without any explicit symbolic reasoning.

The behavior of the actor robot was pre-programmed using hard-coded logic, based on its personal world view. The observer AI was represented as a deep neural network that receives the overhead image as input and produces a prediction of the resulting actor behavior as output. Each input scene is a 64 × 64 pixel image. The output envisioned by the observer is also a 64 × 64 pixel image that depicts the actor’s behavior as a motion smear and removing consumed food circles, if any.

## Network architecture

The architecture of our network is a fully convolutional multi-scale vision prediction (image-to-image) network, comprising 12 fully convolutional layers with 609,984 neurons and 570 K parameters. Such networks are often used for predicting the next frame in a video. However, instead of predicting the next frame in a series of frames, we required the network to predict the last frame based on an image created by integrating the first half of the frame sequence (for the first three policies of the actor robot) or just the first frame (for the fourth policy of the actor robot). This is akin to asking a person to predict “how the movie will end” based on the opening scene.

Specifically, our network comprises two main parts: an encoder network and a multi-scale decoder network. The encoder network is composed of several convolutional layers where each convolutional layer is followed by a batch normalization layer and a ReLU non-linear activation function. Such a unit is denoted as a whole as “Conv” block in Fig. 3B. It has been demonstrated^13,54^ that a multi-scale convolutional network is useful for refining coarse feature representations to higher resolution.

Inspired by these prior works, we used similar multi-scale prediction networks within the decoder network. Each input image is fed into a “Pred” block which is a convolutional layer followed by a transposed convolutional layer and a Sigmoid non-linear activation function to generate a sub-sampled predicted feature map.

This predicted feature map is then concatenated with the output from a “Deconv” block to be fed into the next stage. The output from each stage is sent into both the “Deconv” unit and the “Pred” unit to get the feature predictions of the next stage. By the end of the decoder network, the “Deconv” will generate the final output which has the same size as the input image. Similar to the “Conv” unit, each “Deconv” block in Fig. 3B is composed of a transposed convolutional layer and a Sigmoid non-linear activation function. We minimize a Mean Squared Error loss on pixel values for training our network. We show all the parameter settings of our architecture as well as training process in the Supplementary Material S4 Deep Learning Architecture as well as provide open source access to the code and data used in these experiments.

## Results

**Figure 4:**
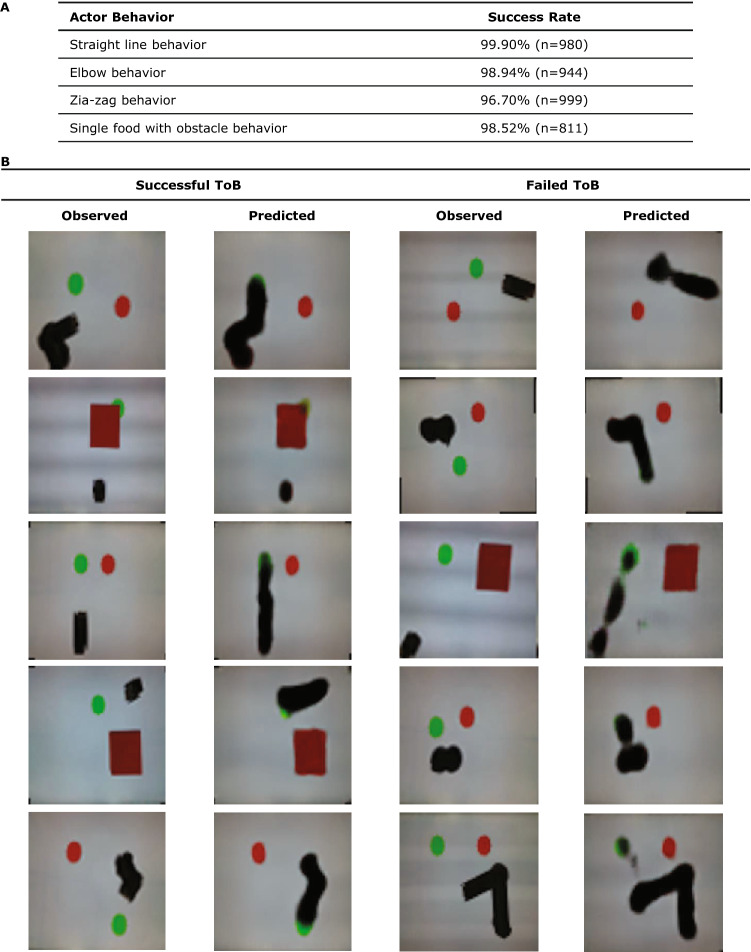
Success rate of observer AI and Physical scenes and outcomes envisioned by the observer. (**A**) We report the successful rate of the observer AI for each type of behavior during testing. Noted that all the behaviors of the actor robot are seen during training together and no other information is given except the single image frame. Our observer AI achieves a 98.5% success rate on average across all four types of behaviors of the actor robot. (**B**) The first column and the third column show some sample scenarios involving the actor robot, one or two green circles, and a square obstacle. The second and the fourth column show the outcome as envisioned by the observer. The left two columns (Success) show successful vision, whereas the right two columns (Failure) show failed envision.

The observer AI was trained using 2400 input–output image pairs recorded experimentally, of which 20 percent were used for testing. The input image is a single frame with the projection of the first half of the trajectory of the actor robot, and the output image is the final frame with the project of the entire trajectory of the actor robot. Both the training and testing data were balanced across all four types of behaviors of the actor robot. Each original image pair was expanded into 10 additional cases using random rotation and symmetry transformations with 0.5 probability of each, in order to augment the training set and testing set. The augmentation was done after splitting into training and testing set so as to not cross-contaminate the training and testing datasets. The resulting performance reported in Fig. 4A was measured on a withheld test set of 1,200 image pairs for each of the four policies of the actor robot.

Figure 4B shows qualitative examples of various raw input scenes as seen by the observer AI, and both successful and failed outcomes it envisioned. When the observer envisions that a green circle is consumed, it clears most of the green pixels. If the observer AI believes that the actor will move, the envisioned the trajectory of the actor robot as a motion smear. We emphasize again that the observer has no explicit notion of food, obstacles, actions, coordinated, position, policies, line of sight etc. It only sees an image of the world and envisions an image of the future. The observer did not know which policy was being employed by the actor.

The proposed visual Theory of Behavior was considered experimentally successful if and only if the scene envisioned by the observer had correctly erased the food consumed by the actor, and also correctly visualized the actor’s motion trajectory. To evaluate whether the observer has successfully foreseen the high-level behavior of the actor robot, we automatically processed the images produced by the observer using classical machine vision techniques. We extracted both the largest contours of the ground truth and predicted trajectory, as well as the position of the food circles using their colors measured from the ground truth image. We calculated the shortest distance from all the points on the contours to the center of the green food (target), we define this value as *D*_*target*_.

In 6.65% of the cases, the machine vision algorithm used to evaluate success or failure automatically was unable to reliably determine the contour or the position of the foods in the ground truth images, and therefore was unable to determine whether or not the behavior was “successful”. These cases were largely caused by a particularly short length of the trajectory leading to situations where the robot already covers a substantial part of the food due to the angle of the camera view. These cases were automatically omitted from the statistical analysis.

For the remaining 93.35% of cases where success or failure can be determined reliably, we compute the success rate by checking if the envisioned actor location is within one robot-diameter from the real actor position. In other words, for each predicted output, if *D*_*target*_ is smaller or equal than the diameter of the Actor robot, we mark that prediction as a success. Otherwise, we mark it as a failure. Then the success rate is calculated by dividing the total number of successful predictions by the total number of predictions. Each individual policy is evaluated separately during testing. However, the category of policies is never used during training. Supplementary Material S3. “Video processing” describes more details about the data processing pipeline. The precise process by which the accuracy was calculated is described in Supplementary Material S5 “Results”.

As shown in Fig. 4A, our observer AI is able to achieve a 98.52% accuracy on average across all four types of behaviors of the actor robot, without knowing in advance which policy will be used. Combing with the qualitative results shown in Fig. 4B, we show that our observer AI is able to understand and predict the behavior of the actor robot without any symbolic representations.

## Handling ambiguity in visual behavior modeling

An interesting question is whether the observer can handle ambiguity. For example, ambiguity may arise from stochastic behavior of the actor or from sensory noise. To test this, instead of integrating the first half of the trajectories of the first three policies of the actor robot into one single input image, we only give the first image as the visual input for all four policies to the observer AI. Now the training input becomes just a single frame from the initial scene, and the output to be predicted is a single image that should integrate all future frames from the first frame to the last frame, across the entire yet-unseen trajectory of the actor robot.

Under this setting, there are multiple possible future behaviors of the actor robot when given only single starting frame. The question is not whether or not the prediction will be correct (as it is impossible), but rather how with the observer represent the ambiguity.

**Figure 5:**
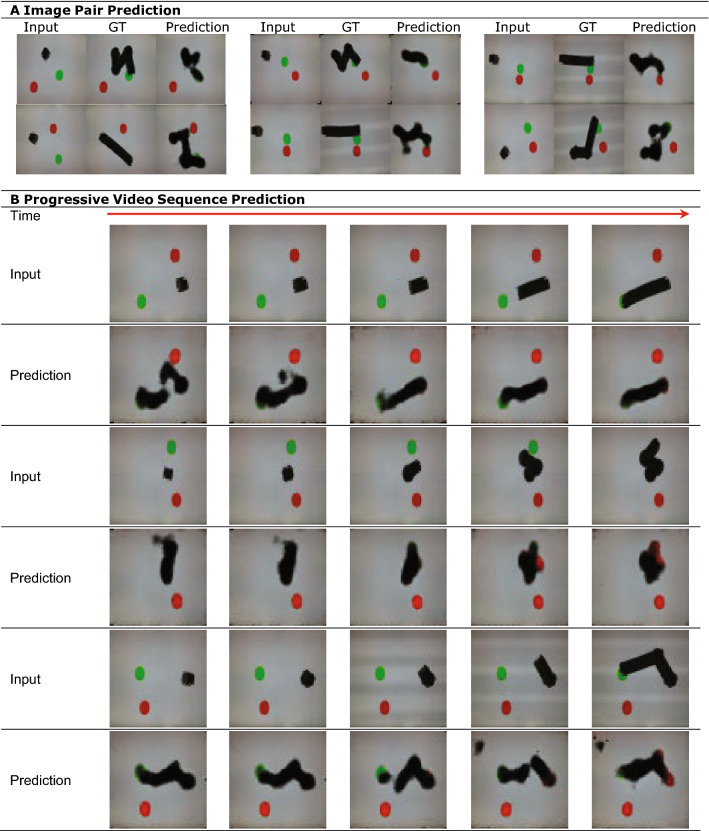
Handling ambiguity. This observer AI is trained only on the first frame as input. Therefore, there are multiple possible future trajectories possible based on only this input frame. As shown in (**A**). Image Pair Prediction, the observer handles this ambiguity by outputting one of the heuristic behaviors or as a blurry trajectory. Although our observer model is trained using start and end image pairs (with integration of all the past frames), the observer can be used in an online fashion during evaluation. (**B**) Progressive Video Sequence Prediction shows the prediction results using our model across multiple time stamps.

Figure 5 shows that the observer AI handle the uncertainty by generating blurry, foggy images.

We also wanted to investigate if the level of blurriness is reduced as the observer is given more frames from the activity of the actor, thereby reducing the uncertainty. We fed the observer input image that integrated increasingly longer series of frames up to the current time stamp. In Fig. 5, we show the sequence of input and output images subject to uncertainty.

## False belief test

**Figure 6:**
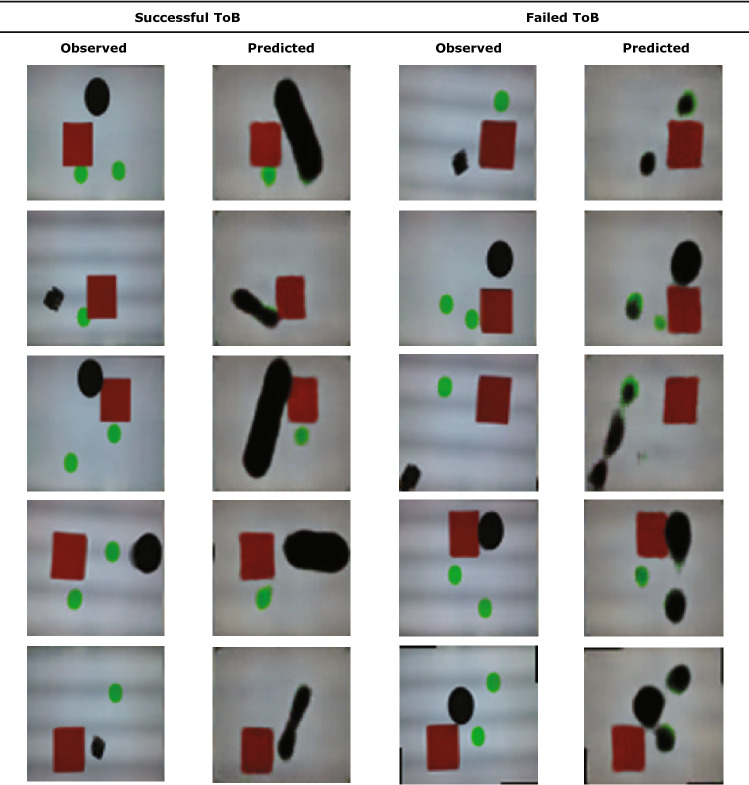
Physical scenes and outcomes envisioned by the observer. The first column and the third column show some sample scenarios involving the actor robot, one or two green foods, and a square obstacle. The second and the fourth column show the outcome as envisioned by the observer. The left two columns (Success) show successful vision, whereas the right two columns (Failure) show failed or blurry envision.

To ensure that our proposed Visual Theory of Behavior is consistent with protocols commonly practiced in studies of Perspective Taking or Theory of Mind, we further designed our experiments to include a false belief test. In this case, the actor robot has one simple, pre-programmed (hard coded) behavior. The actor robot always moves towards the nearest green circle that it can see with a straight line of sight. When two circles are visible, the actor will move towards the closest circle. However, if the closest circle is hidden behind an obstacle, such as a red box, then the actor will not see the green circle and will therefore not move towards it. Instead the actor might move towards a different circle that is visible. Similarly, Fig. 6 shows the qualitative examples of the visual observations as well as predictions envisioned by the observer AI. The observer AI was trained using 600 input–output pairs which then are augmented to 6,000 image pairs in the same way as indicated above.

There are mainly two types of sub-behaviors under this single policy of the actor robot. We call it a “food visible” case when the actor robot perceives the physically closest food and hence moves towards it. Alternatively, we call it a “food obscured” case when the actor robot is unable to see the physically closest food due to the occlusion by an obstacle, and only consumes the visible food which is further from it.

**Figure 7:**
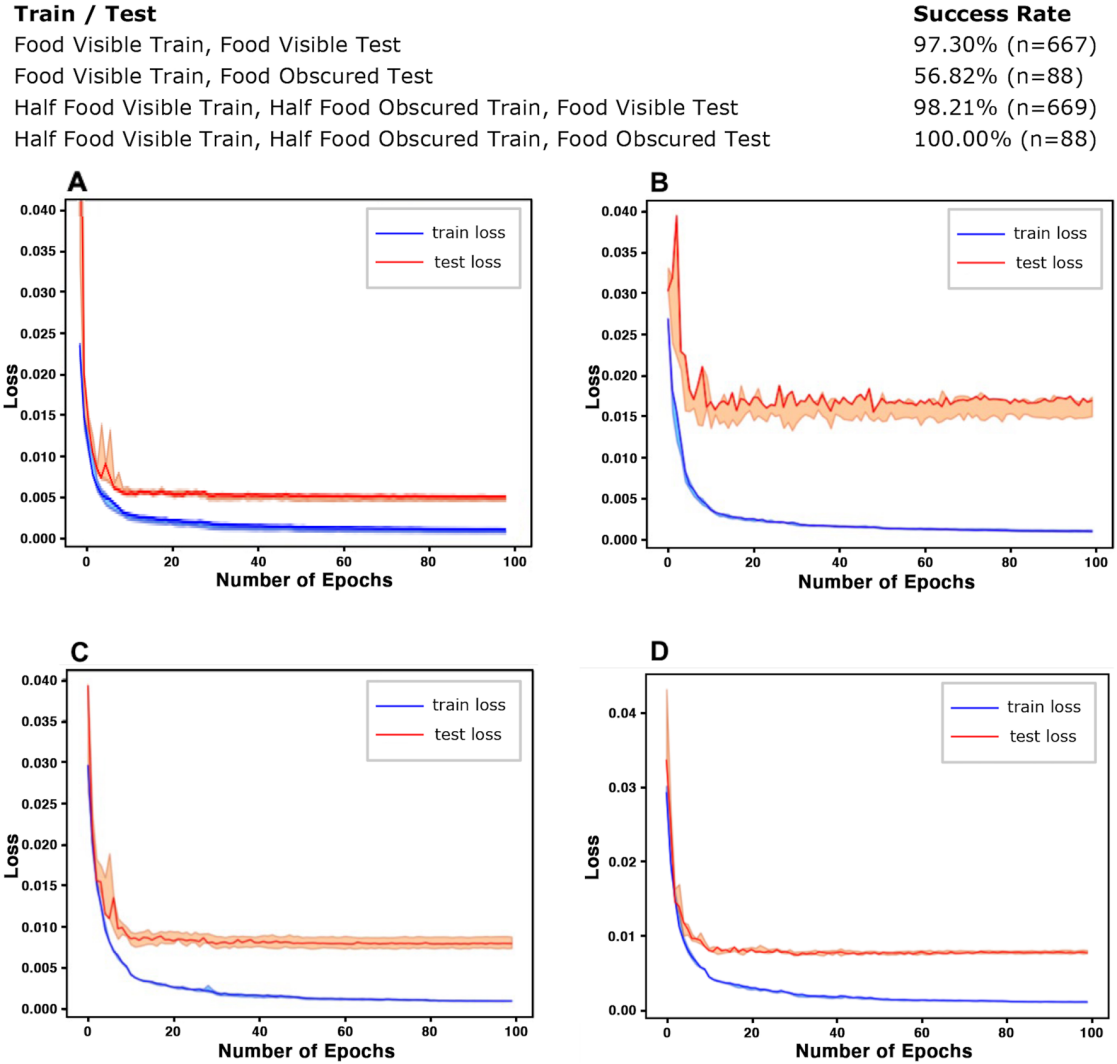
Training and testing of the observer and corresponding success rate We first gathered training and testing data by randomly placing the actor, two green food items, and the obstacle (Table, **A**). We also collected “obscured” test cases where we deliberately placed the closest food to where it is not visible to the actor (Table, **B**). Higher success rates were achieved by balancing the training data with half “visible” data and half “obscured” data (Table, **C** and **D**). Learning curves across all four above scenarios are shown. Error bars are presented to show experiment results across three different random seeds used in both data splitting and network training.

This experimental design can be seen as a false belief test. In the first step, we train the observer agent only with the “food visible” cases where the observer only sees the actor moves towards the closet food. However, the actual policy of the actor robot is to consume the closest food *it can see*. Hence, there should be a mismatch of understanding of the policy between the observer and the ground-truth policy of the actor. Indeed, our results in Fig. 7 shows that if we test the observe model with only “food visible” cases, the observer AI was able to correctly envision the scene with 97.3% accuracy. However, if we test the observer model with only the “food obscured” cases, the observer AI’s accuracy drops to 56.82%. This means that the observer trained only on food visible cases fails to notice the difference of the perspective between itself and the actor robot.

After we balanced the training dataset by including 50% “food visible” cases and 50% “food obscured” cases, the results change. We then test the trained observer model with “food obscured” testing set again. As shown in Fig. 7, the observer AI achieved 100% accuracy on all the “food obscured” testing set and even further improves the “food visible” testing accuracy to 98.21%. This statistically significant improvement in the performance can be interpreted to imply that it is necessary to teach perspective taking to the observer agent under our setting by exposing it to both food visible and food obscured examines.

**Figure 8:**
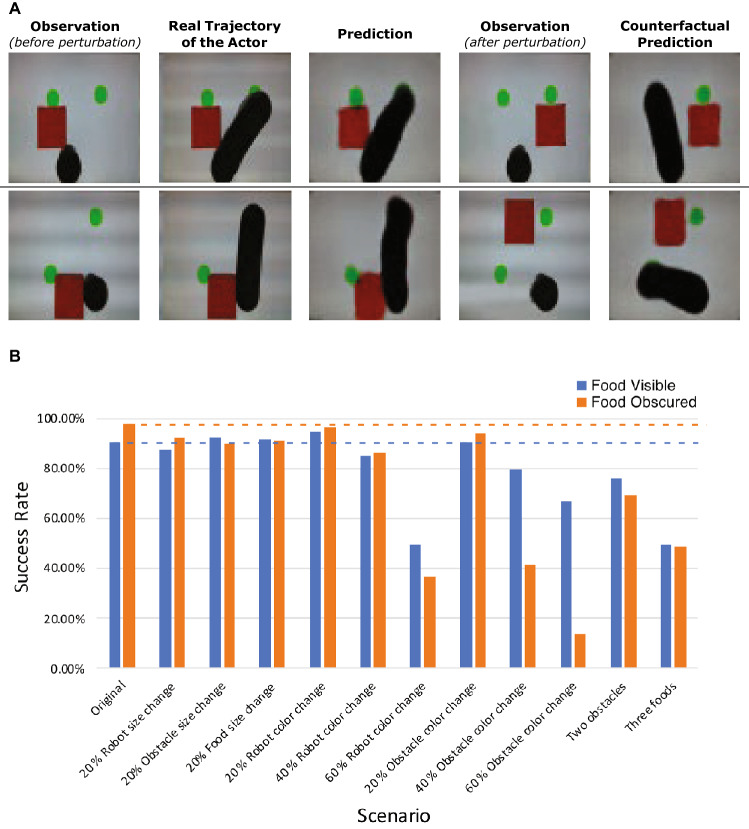
Counterfactual perturbation and prediction sensitivity. (**A**) The first column shows the original observation of the observer and the third column shows the prediction from the observer. The second column is the real trajectory of the actor robot after releasing it. We then move the obstacle to a different location from the first column. The resulted new observation of the observer is shown in the fourth column. The last column shows the counterfactual prediction after seeing this new observation. Both examples show the observer changes its prediction after the physically closest green dot becomes visible from being obscured by the obstacle. (**B**) Our model is able to give accurate prediction when the sizes of different objects as well as actor robot is changed. However, the observer model performs worse when the color is changed by 40%, and eventually breaks when the color is changed by 60%. The size change in these experiments is limited by the arena size (128 $\times$ 128 pixels).

Figure 8A presents qualitative visualizations when we perform counterfactual perturbation to the observation of the observer agent, under “food obscured” cases. Specifically, by moving the obstacle from blocking the physically closest food to blocking the physically farther food, we observe that the observer changes its prediction accordingly, which further validates that the observer recognizes the perspective differences between itself and the actor robot.

To further investigate to what degree our observer network is able to model behavior of the actor robot under various unseen changes in the environment, we replicate the exact setting and data processing pipeline from the real world in simulation and perform a systematically evaluation. Specifically, we first vary the size of the robot, the obstacle and the foods in the “food visible” testing data and “food obscured” testing data respectively. We only vary one element at a time. We then gradually change the color of the food and the obstacle to each other. Finally, we add one additional food and one additional obstacle respectively during testing.

The results are summarized in Fig. 8B. Our observer model is still able to give accurate prediction when the sizes of the objects are changed, but performs worse when the color is changed by 40% and finally breaks when the color is changed by 60%. This suggests that our observer model does not heavily rely on size of the entities to model the behavior, but it remembers the color information to some extent to assist the prediction. Specific results for each group can be found in Supplementary Material S5. Results.

## Discussion: (how this supports the hypothesis)

The ability of the observer to visualize both the actor’s behavior and its consequence suggests that the observer was able to represent, at least implicitly, the mental state of the actor. As in any ToM experiment (human or robotic), it is impossible to prove with certainty whether the observer fully models the actor, or whether it merely appears to do so.

An inevitable question is why would envisioning the outcome as an image be preferable to explicitly reasoning about the outcome. After all, symbolic reasoning can be much faster and more efficient than visual reasoning. Also, visual reasoning involves analyzing over 12,000 input values and generating over 12,000 output values. This seems to be an overkill for a simple task that involves just a few nested logic conditions.

We argue that visual reasoning carries several key advantages over logical reasoning. First, it sidesteps the symbol grounding problem ^55–58^ that has plagued AI for decades. The observer does not need explicitly understand the notion and properties of discrete entities, trajectories, conditional rules, geometry or physics, because it deals only with pixels. Second, we believe that image processing ability is a more primitive capability that would emerge earlier on an evolutionary time scale, compared to symbolic reasoning which likely evolved later and may require a developed neocortex.

Visual representation of actor behavior can also naturally accommodate uncertainty, both with respect to the actor’s actions as well as with respect to the outcomes of those actions. This uncertainty is reflected as blurry images or one of the heuristics of the potential behaviors, as seen in Fig. 5. Here, the predicted image occasionally envisions the actor as appearing in multiple places simultaneously or as a blur.

The observer could also readily take actions based on a visual prediction, using pre-adapted visual processing apparatus that were evolved for responding to real scenarios. For example, a pray that can envision a predator’s action may be able to respond more rapidly using the same mechanism it would use to escape a real predator which it can literally see. Compare that to a pray that must first convert sensory information to symbols, and then reason symbolically about a predator’s action symbolically.

## Conclusions: (limitations, caveats, future work)

We proposed and demonstrated an AI agent can observe an actor robot and foresee its future actions plans before they happen in reality. We demonstrate that this process can occur in an end-to-end manner using only raw camera data as input, without assuming any symbolic reasoning nor any feature engineering, neither in the sensory input nor in the output.

We conjecture that perhaps, our ancestor primates also learned to process a form of behavior prediction in a purely visual form, long before they learned to articulate internal mental visions into language, to others or even to themselves.

There are clear evolutionary advantages to such an ability. For example, our mind’s eye is rapid—it can perhaps see an imminent collision with another person, long before we think about it consciously or verbally^59,60^. This mental image allows us to move out of the way because we can literally see the impending collision before it happens, without resorting to slow symbolic processing metal apparatus.

There are many limitations to this study that would require additional future research. For example, we allowed the observer to have full overhead view. In practice, the observer typically only has a first-person view or partial information, making inference of the actor’s view more difficult. Second, it would be interesting to explore whether visual reasoning can handle more complex actor logic as that presented by Rabinowitz^8^. Moreover, how to build agents that can be aware of the behavior and perspective of other agents in multi-agent scenarios where interaction between multiple agents becomes important is an exciting next step for further works.

The “visual foresight” we demonstrated here is primitive, but we suggest it may be key to understanding many forms of social behaviors in humans and primates. The ability of a machine to predict actions and plans of other agents without explicit symbolic reasoning, language, or background knowledge, may shed light on the origins of social intelligence, as well as pave a path towards more socially adept machines.

## Supplementary information

Supplementary information.

Supplementary information.

## Data Availability

All the data and code are publicly available at https://github.com/BoyuanChen/visual_behavior_modeling.
