# Supplementary material for: Visual behavior modelling for robotic theory of mind
Source: Sci Rep. 2021 Jan 11;11:424. doi: 10.1038/s41598-020-77918-x (PMC7801744; doi:10.1038/s41598-020-77918-x)
Supplement: Supplementary file 1 — Supplementary information. [file 41598_2020_77918_MOESM1_ESM.pdf]

Supporting Material for
Visual Behavior Modelling for Robotic Theory of Mind
Boyuan Chen*, Carl Vondrick, Hod Lipson

Contents :

S1. Experimental setup
S2. Robot details
S3. Video preprocessing
S4. Deep learning architecture
S5. Results

S1. Experimental setup

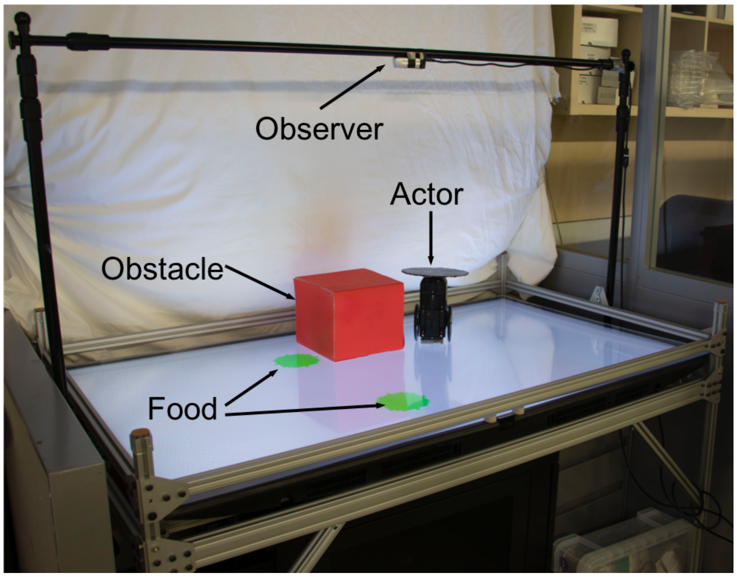

Fig. S1 Experiment platform setup. We built an experimental platform for data collection and evaluations. An Actor robot navigated on top of a TV display where different green dots or red dots are displayed. An Observer watches the scene through a bird-view camera.

We performed all of our experiments on the hardware setup shown in Fig. S1. In order to automatically generate and display the target food and other foods on the background, we used a flat panel display. This display has a screen size of 57.5 inches by 33 inches and 2160P (4K) resolution. We then connected the display to a computer to send auto-generated background maps.

The Observer machine is an Alienware-15 laptop connected to a top-view camera to acquire the scene videos as seen from above. The camera used was an Intel RealSense Camera (D415) with up to $1280 * 720$ resolution. Higher resolutions tended to capture refresh patterns.

We set up a camera support structure (shown in black over the display) and a display support structure (shown in silver below the display) respectively to attach both the top-view camera and the display together. The Actor Robot navigated around on top of the display surface. The robot was controlled by a master computer through Wi-Fi network. To protect the surface of the display while running the robot, we place a clear acrylic sheet on top of the display.

We now describe the steps that we used to conduct our experiments. First, we define each trajectory as the full motion path from the moment the robot starts to execute its internal policy until it finishes.

For each such trajectory, we place the actor robot at a randomly-generated position on the background display. Then the display shows a background image that has various configurations, depending on the type of the designed environments. If there is an obstacle in the pre-defined environment, a randomly generated obstacle position will be displayed and we place a red cardboard box at the corresponding location, to serve as a real obstacle.

In order to avoid the possibility of the observer determining the robot orientation from the direction its wheels are pointing, we covered the robot with a black cylinder. This is why the robot looks like a black circle in the videos. We show some of examples of the initial state at the beginning of each policy in Fig. S2.

After the initial setup, the Actor will start to execute its internal policy, while the Observer starts to record the video for the current trajectory through the bird-view camera until the Actor finishes. We then repeat the above steps with different positions of the foods and the obstacles, if applicable, to collect our dataset.

S2. Robot details

We designed a 3D printed robot for our experiments. There are several practical challenges to address in this experiment. First, robot must be small enough to have room to maneuver on the display. Second, the battery of the robot requires sufficient power capacity to enable extensive data collection continuously. Such scalability is extremely important for data-driven approaches.

To overcome these challenges, we present our design of the Actor robot. The robot is a two wheeled robot with fully 3D-printed body and off-the-shelf electronic components. We show different views and dimensional information of the robot in Fig. S3. We will also open source both the hardware design (CAD files for 3D printing, links for available

parts inside the robot and assemble manuals) and software packages for this robot upon the paper is accepted. In Table 1, we show the key parameters of the Actor robot for references.

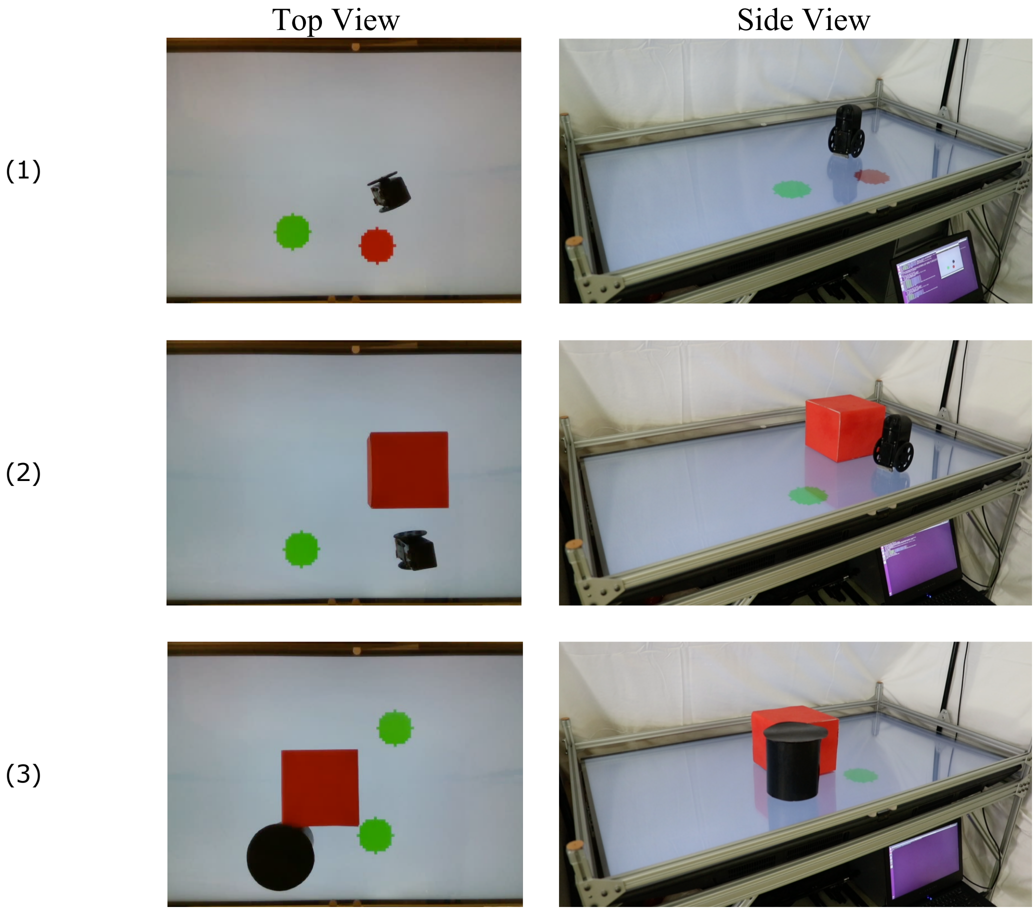

Fig. S2 Examples of initial setup for each actor policy. Each row shows a pair of images taken from different angles while the actor robot performs various policies. The left column is taken from the top view camera and the right column is taken from a side angle to show a "1st person view". (1) The Actor robot always navigates towards the green food and ignore the red food. (2) The Actor robot always go to the visible green food. If there is no green food visible (e.g., the green food is occluded by some obstacle), the Actor robot will stay in put. (3) The Actor robot always pursues the closest food it can perceive.

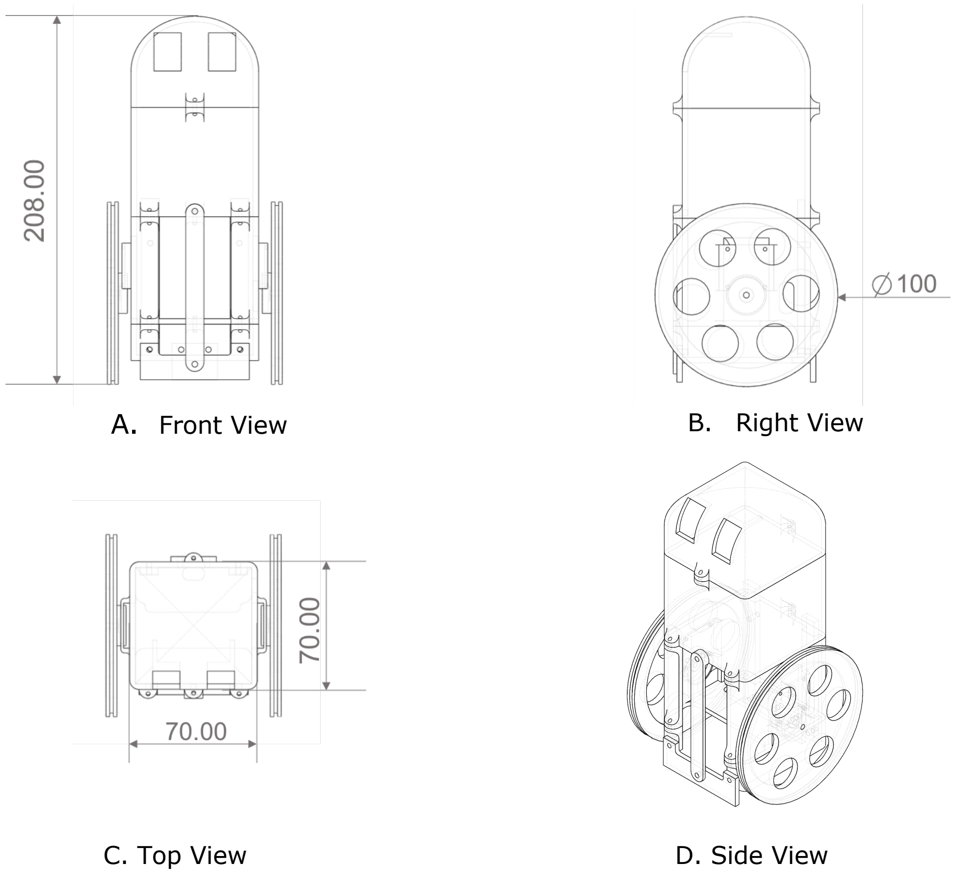

Fig. S3 CAD model of the Actor robot. We use this CAD file to 3D print the whole body of our Actor robot. Different views (unit: mm) have been shown in each figure along with important dimensional parameters.

	Specifications	
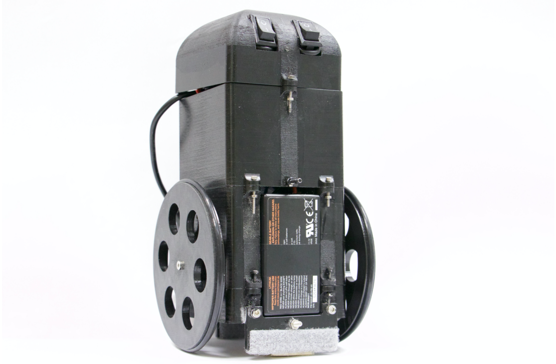	**Basic Capacities**	Navigation, Remote control, easy to attach various sensors such as camera, microphone etc.
	**Size**	See Fig. S3.
	**Weight**	654 g
	**Speed Range**	0 ~ 30 cm / s
	**Accuracy (Straight Line):**	Approximately 1.5 cm off after running with 15.5 cm / s for 1 second (before calibration)

Table 1 Key parameters of the Actor robot.

S3. Video preprocessing

With the intention of creating a pure visual theory of mind model, we preprocessed the recorded videos into two compound images. The steps involved in the video collection are described in the previous section. We now discuss the video processing steps. Fig. S4 provides an overview of the entire processing pipeline. In essence, a compound image is

created by taking the minimum value of each pixel over an entire video. This compresses a video into a single image. We assume standard RGB encoding,

We denote the video frames $I_{i,0}$, $I_{i,1}$, …, $I_{i,T}$ as corresponding to the frames of the ith trajectory where T is the total number of frames in the current trajectory. We provide the Observer one single initial frame image as the input and expect the Observer to predict a single image that describes both the future trajectories of the Actor and the goal of the Actor.

To do so, we stacked all the frames from $I_{i,0}$ to $I_{i,T}$ by saving the smallest pixel values across of these frames to form one single output image. The input is just one 1st initial frame image. This pair of input and output image is further used to train our visual theory of mind model in a supervised way. We show some example input frames and output images in Table 2.

Intuitively, stacking images like this is trying to project all the past waypoints of the Actor onto one single image without losing the background environment information. Hence, the output images have the whole trajectory of the Actor robot among the entire process. It is worth to note that we do not specifically track the position of the robots nor hand-crafted any image features to achieve this.

We collected 600 real world videos. We preprocessed these videos using the aforementioned method which gives us 600 input and output pairs of images. Furthermore, we augment 500 of them with 0.5 probability of being flipped right to left, and 0.5 probability of being flipped up and down, to get 5,000 training image pairs. Using the same augmentation pipeline, we augment 100 of the images to get 1,000 testing data pairs. The code for the whole preprocessing pipeline will be made available on GitHub upon the paper is accepted.

Policy Description	Input	Target Output
**Straight Line Policy:** The Actor always goes to the green food in straight line.	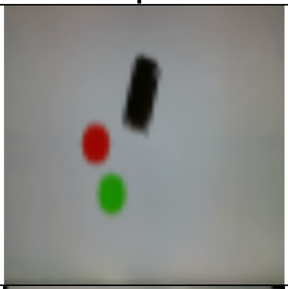	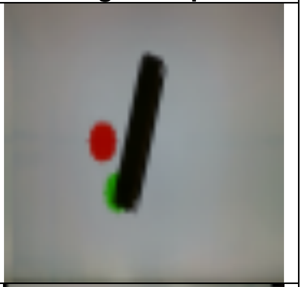
**Elbow Policy:** The Actor always goes to the green food by first going to a control point and then navigating to the green food in straight lines.	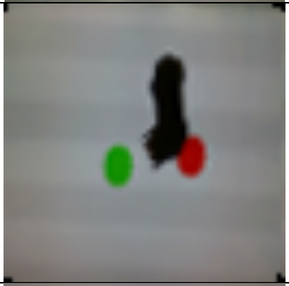	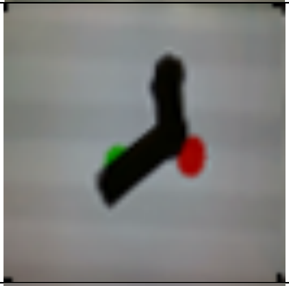
**Zig-Zag Policy:** The Actor always goes to the green food by first going to two control points subsequently and then navigating to the green food in straight line.	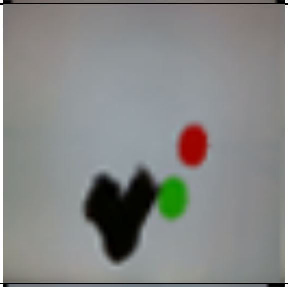	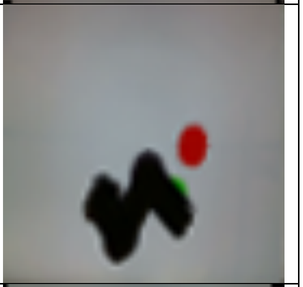
**One Food Obstacle Policy:** The Actor always goes to the green food if it is visible to the robot. Otherwise, the Actor robot will not move. (The red cube is the obstacle and the robot is not able to see through it.)	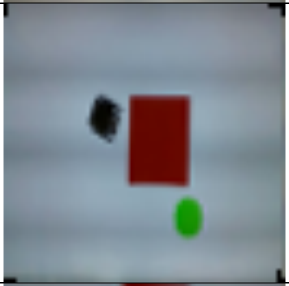	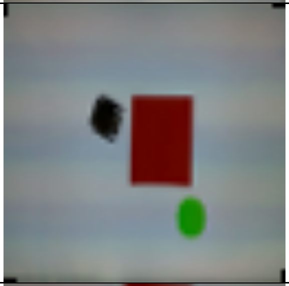
**Two Foods Obstacle Policy:** The Actor always goes to the closest green food it can see in a straight line. (The red cube is the obstacle and the robot is not able to see through it.)	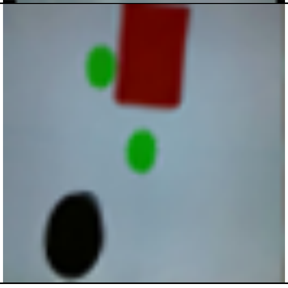	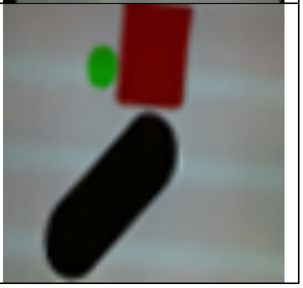

Table2: The descriptions of the Actor policy.

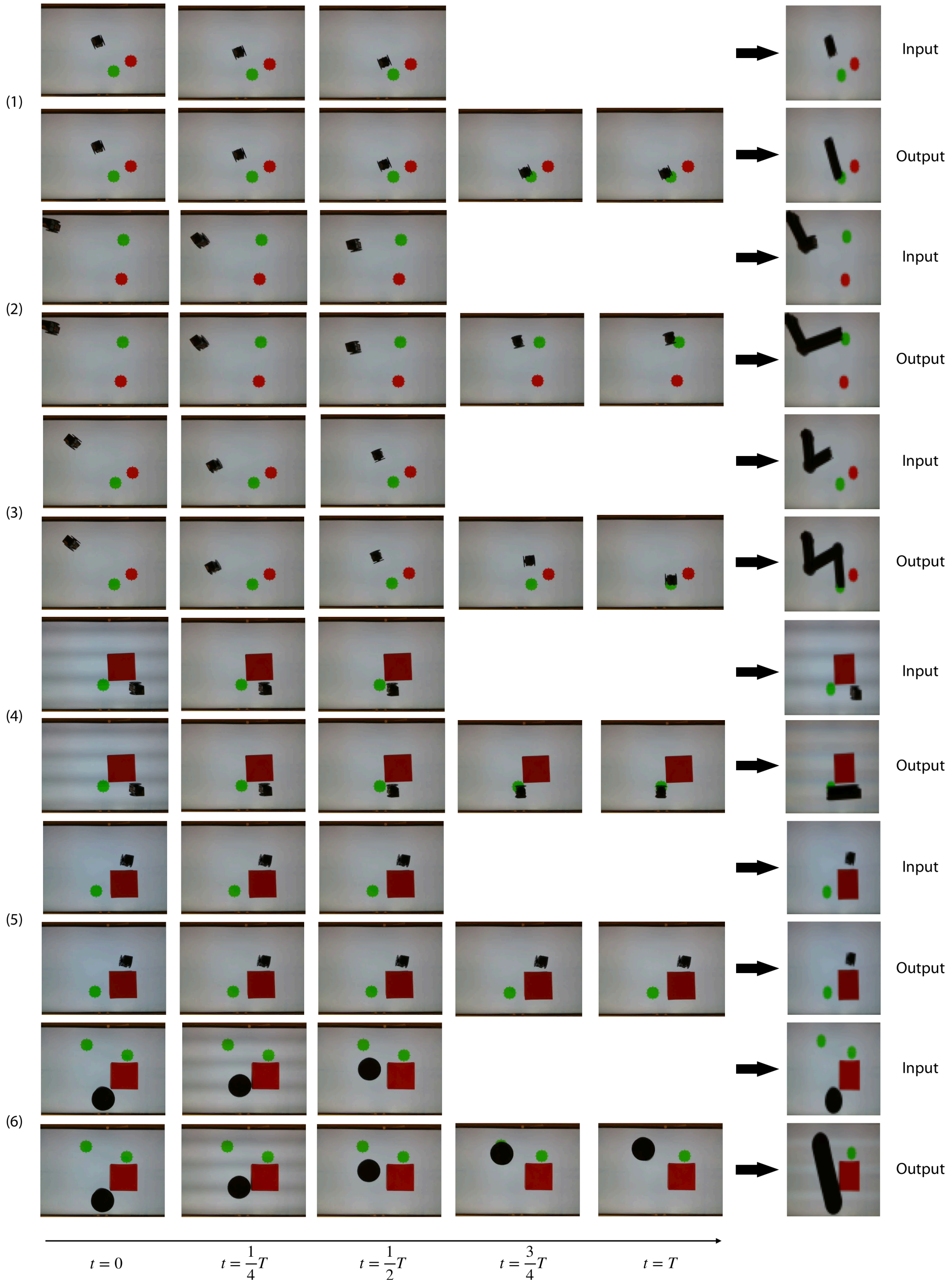

Fig. S4 Examples of data preprocessing pipeline of straight line policy, "elbow" line policy, "zig-zag" line policy and one single food with obstacle policy. Every two rows correspond to one preprocessing pipeline. In the 1st row, all the frames in the first half of the video are stacked together to generate one single input image. In the 2nd row, all frames on the current video are stacked together to form one single target image. Here we only show three frames and five frames for each row for illustration purposes. In reality, we process all of the obtained video frames. As we can see in the last column, each single image contains all the information of the current environment and the encoded past trajectory of the Actor robot.

S4. Deep learning architecture

We designed a multi-scale fully convolutional encoder-decoder network to serve as our image prediction network. An overview of our network architecture is shown in Fig. S5. Our network takes one single RGB image as the input and generates one single RGB image as output. Both the size of the input I_{input} and the size of the output I_{output} are 64 × 64 × 3. The network mainly has two parts, an encoder network and a multi-scale decoder network. The encoder network is composed of several convolutional layers where each convolutional layer is followed by a batch normalization layer and a ReLU non-linear activation function. Such a unit is denoted as a whole as "Conv" block in Fig. S9.

It has been demonstrated[13,54] that a multi-scale convolutional network is useful for refining coarse feature representations to higher resolution. Inspired by these prior works, we used similar multi-scale prediction networks within the decoder network. Each input feature is fed into a "Pred" block which is a convolutional layer followed by a transposed convolutional layer and a Sigmoid non-linear activation function to generate a sub-sampled predicted feature map. This predicted feature map is then concatenated with the output from a "Deconv" block to be fed into the next stage. The output from each stage is sent into both the "Deconv" unit and the "Pred" unit to get the feature predictions of the next stage.

By the end of the decoder network, the "Deconv" will generate the final output which has the same size as the input image. Similar to the "Conv" unit, each "Deconv" block in Fig. S9 is composed of a transposed convolutional layer and a Sigmoid non-linear activation function. We show all the parameter settings of our architecture in Table 3. We optimize our network to minimize a Mean Square Error loss. We train our network with Adam optimizer with an initial learning rate 0.01 and batch size 128 for 100 epochs. The learning rate decays by 90% at epoch 10, 30, 50, and 80.

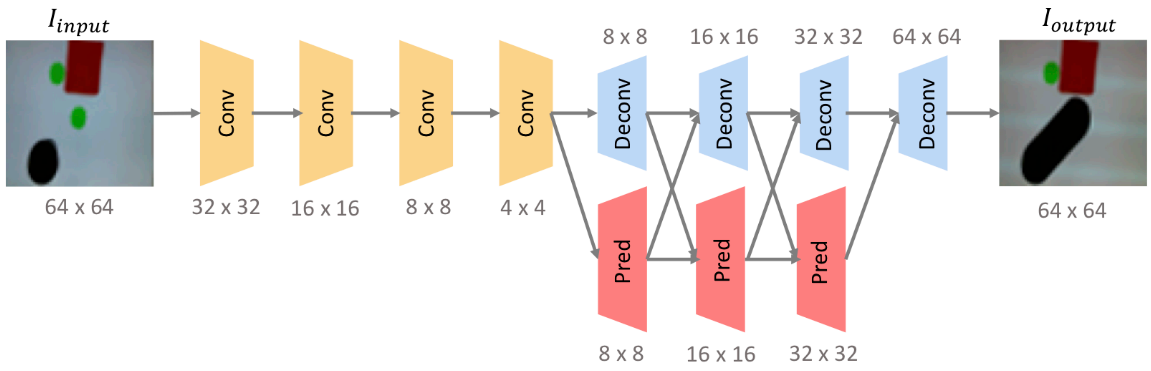

Fig. S5 Image Prediction Network Architecture Our image prediction network is composed of several layers of convolutional layers and deconvolutional layers. At the deconvolutional stage, we utilize multi-scale prediction to maintain high resolution in our output image. We list all of the parameters used in the architecture in Table .

Layer	Kernel Size	Num Outputs	Stride	Padding	Dilation	Activation
Conv1	4 × 4	32	2	1	1	ReLU
Conv2	4 × 4	32	2	1	1	ReLU
Conv3	4 × 4	64	2	1	1	ReLU
Conv4	4 × 4	128	2	1	1	ReLU
Deconv4	4 × 4	64	2	1	1	Sigmoid
Deconv3	4 × 4	32	2	1	1	ReLU
Deconv2	4 × 4	16	2	1	1	ReLU
Deconv1	4 × 4	3	2	1	1	ReLU
Pred3Conv	3 × 3	3	1	1	1	N/A
Pred2Conv	3 × 3	3	1	1	1	N/A
Pred1Conv	3 × 3	3	1	1	1	N/A
Pred3Deconv	4 × 4	3	2	1	1	Sigmoid
Pred2Deconv	4 × 4	3	2	1	1	Sigmoid
Pred1Deconv	4 × 4	3	2	1	1	Sigmoid

Table 3 Parameters of the Image Prediction Network The name of the layer corresponds to the unit in the architecture and the number indicates the sequence of the current layer. We choose to use Sigmoid as our final activation function due to the normalization.

Fig. S6 shows more sample input, target output and predicted output images after training the network. Qualitatively, our predictions generally match the target outputs and can successfully model the policy of the Actor robot and its internal hidden goal. We also show the learning curve of the "hardest" policy across multiple runs in Fig. 8. We will release the full experiment code base on GitHub upon the paper is accepted.

S5. Results

This section describes the evaluation matrix and provides examples of both success and failed results.

To evaluate our methods, we present both qualitative and quantitative evaluation results to inspect whether our proposed algorithm can enable the Observer to forecast the future trajectories of the Actor as well as its final goal.

S5.1 Qualitative Results

In this section, we present the qualitative assessment of our framework. It is noteworthy that our framework does not assume that the observer has any prior knowledge of the environment and the goal of the actor agent. All the operations are performed at the pixel level. In addition, the observer network does not have access to the ground-truth policy of the Actor robot. The main purpose of this evaluation is to determine if our predictive vision model is able to give high-level prediction, and assess if its ability can match basic capacity for Theory of Mind under the same settings.

As shown in Fig. S6, our model provides the desired prediction, which completes the future trajectories of the actor robot and successfully draws the consequences of its actions (consume the green food, if any). Interestingly, in some cases where two balls are very close to each other and the current position of the actor robot is roughly equidistant to the locations of the two balls, it is very hard even for a human to give an accurate prediction.

It should be noted that even in the hardest cases shown as the last section in Fig. S6, our model is still able to perform satisfactorily. In this case, the internal policy of the Actor is to go to the closest food it can see. Since the Observe has a bird-view, it has to understand that the Actor has a different view of the "world" and even if the Observer perceives the closest food to the Actor, the Actor will not consume it because the Actor is not able to see that. Hence, to deliver an accurate prediction of the goal and the policy of the Actor robot, the Observer has to understand such a high-level rule which demonstrates Robot Theory of Mind.

S5.2 Quantitative Results

In this section, we will first describe the evaluation matrix that we used throughout our experiments and then discuss the details about the statistics of our experiments.

In the view of the fact that we do not assume any prior knowledge of the Actor robot, we do not have the exact position of the Actor robot both in groundtruth and predicted frames. Another important question to ask is: how are we going to evaluate whether the model has successfully predicted the high-level goal of the Actor? Traditionally, researchers use pixel-wise accuracy to measure the accuracy of image prediction. However, this matrix does not apply in our case since one does not need to accurately predict what is the color of the pixel by the end of the trajectory to model Theory of Behavior but the high-level description of the policies and goals. Therefore, we present a matrix that we call Goal Directed Error to evaluate our algorithm.

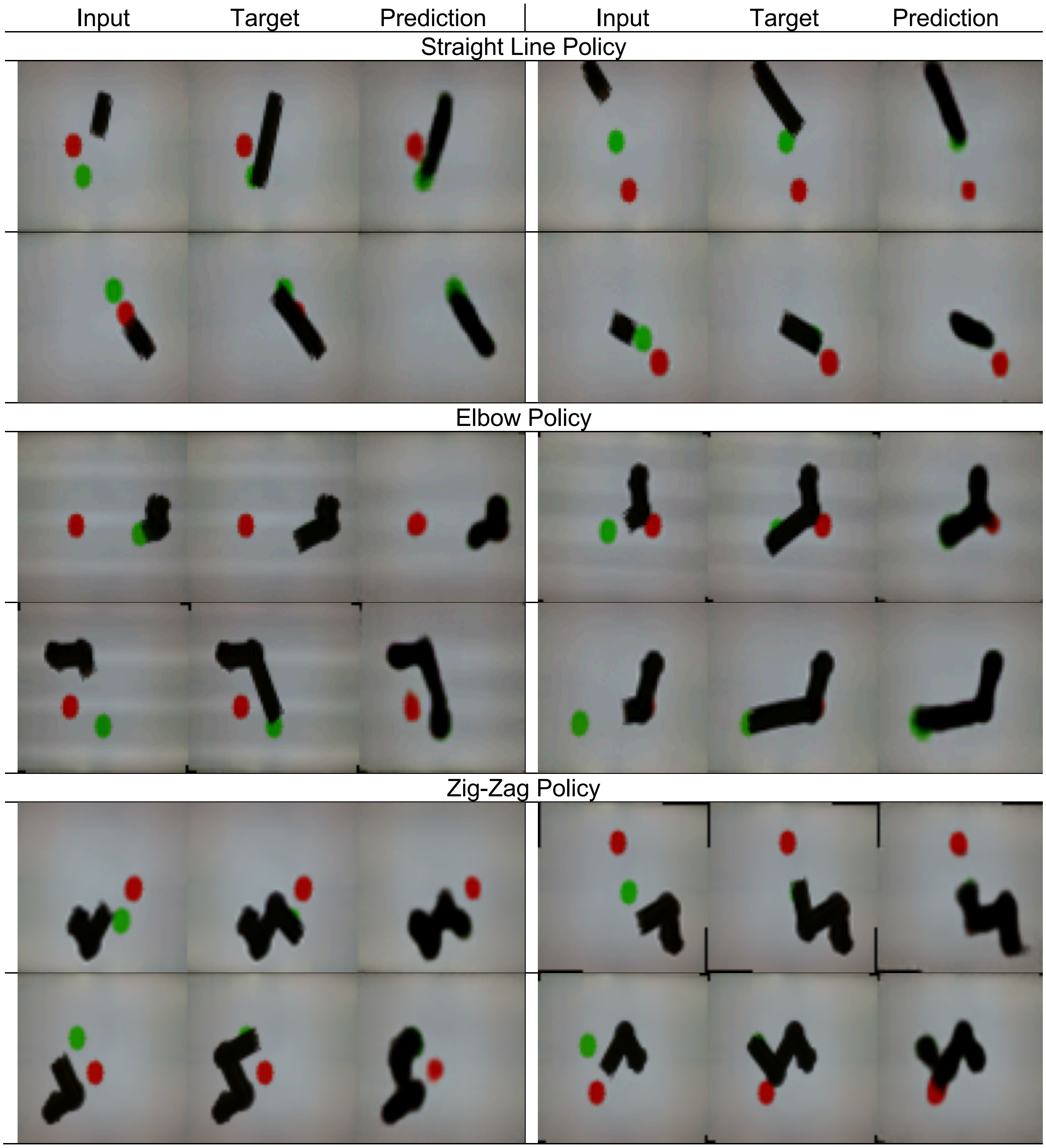

Fig. S6 Qualitative Results We show some examples of the prediction results from our Observer network. Each group of three images represent one example. The 1st image in each group is the input image shown to the Observer network and the 2nd image in each group is the target output of the Observer network. We get the 2nd image by recording the real motion of the Actor robot. The 3rd image is predicted from our Observer network and it should match the 2nd image if the Observer is able to successfully infer the future trajectories and the goal of the Actor robot.

Specifically, we process the images to extract the largest contour of the ground truth and predicted trajectory in the color of trajectory. At the same time, we use the same way to extract the position of the red food and the green food. By calculating the smallest distance from all the points on the contours to the center of the green food (target), we

define this value as D_{target}. We discard the example pairs if the algorithm is not able to find the contour or the position of the foods in the ground truth images. These cases are largely caused by the short length of the trajectory and the robot already covers most part of the food due to the angle of the camera view. With these values, we compute the successful rate as following: for each predicted output, if D_{target} is smaller or equal than the diameter of the Actor robot, we mark this prediction as success. Otherwise, we mark it as failure. Then the successful rate is calculated by dividing the total number of successful predictions by the total number of predictions.

**Policy Type**	**Success Rate**
Straight Line Policy	99.90% (n=980)
Elbow Policy	98.94% (n=944)
Zig-zag Policy	96.70% (n=999)
One Food Obstacle Policy	98.52% (n=784)
Two Foods Obstacle Policy (Visible Train, Visible Test)	97.30% (n=667)
Two Foods Obstacle Policy (Visible Train, Obscured Test Only)	56.82% (n=88)
Two Foods Obstacle Policy (Half Visible Train, Half Obscured Train, Visible Test)	98.21 (n=669)
Two Foods Obstacle Policy (Half Visible Train, Half Obscured Train, Obscured Test Only)	**100.00** (n=88)

Table 4 Success Rate

We also show some of the successful examples and failure cases using our evaluation matrix in Fig. S7 and Fig. S8. Note that even in the failure cases, some of them still look convincing that the model is learning some high-level behaviors. In Table 4, we show the statistics of the evaluation results. As shown in the table, our method achieves high accuracy on the presented task.

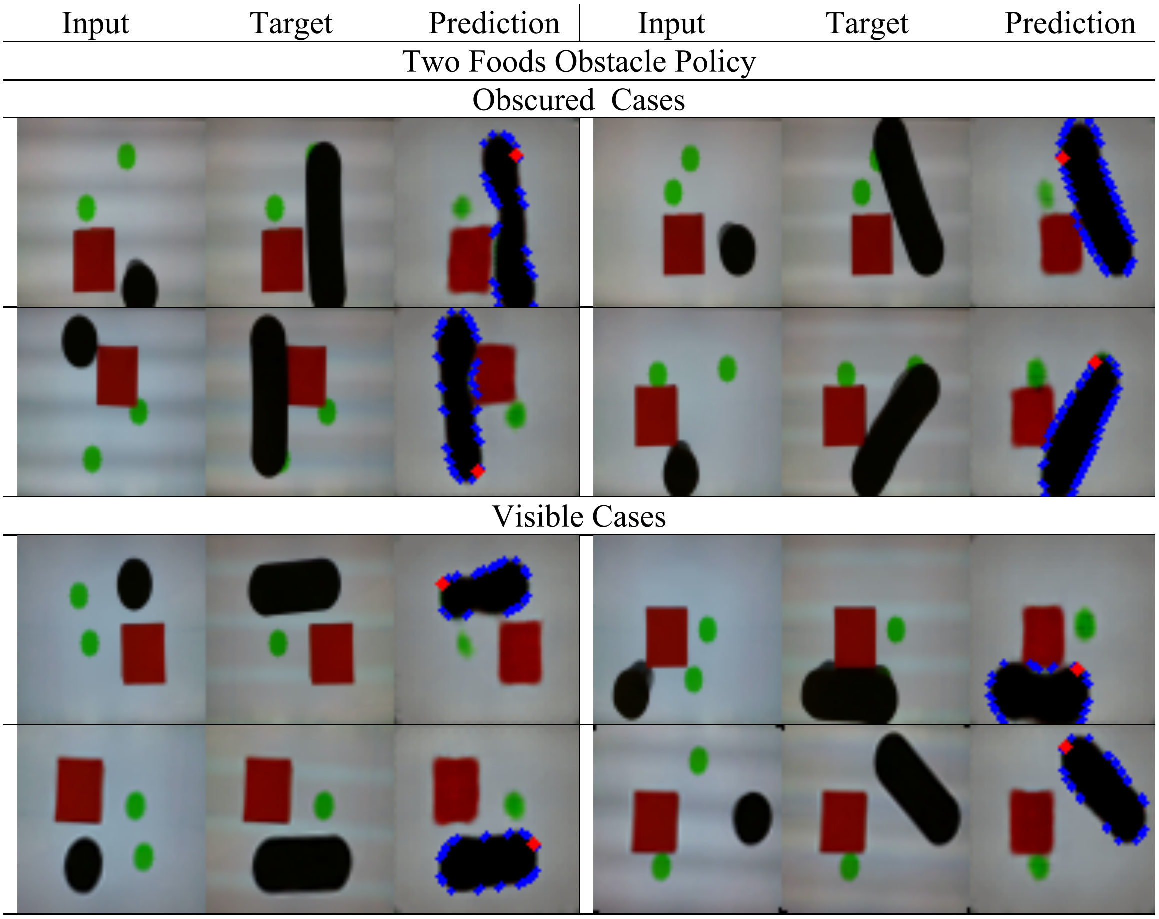

Fig. S7 Successful testing examples. To evaluate our method, we find the largest contour on the predicted image and examine the distance between the closest point on the contour to the center of the green food. We show the founded contour in blue dots and the closest point as a red star. In the cases where the robot cannot perceive the green food due to occlusion, the detected position of the contour should be same with the original input position within a reasonable limit.

Two Foods Obstacle Policy
Obscured Cases
N/A
Visible Cases

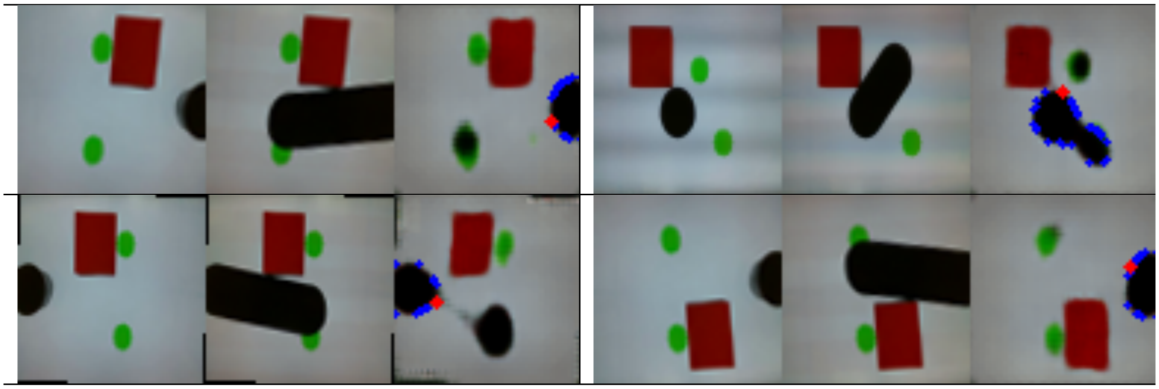

Fig. S8 Failure testing examples. Here we show some failure cases based on our evaluation. Most failure cases are caused due to the disconnected prediction of the future trajectories.

S5.3 Perturbation Results

We vary the size and colors of different entities in the scene to further study to what degree our observer network is able to model the behavior under unseen scene changes. Full results of this experiment are shown in Fig. S9 and Fig. S10. The color is changed by following the color palette at: https://www.schemecolor.com/green-to-red-gradient.php. We also show the visual examples for each variant in Fig. S11, Fig. S12, and Fig. S13.

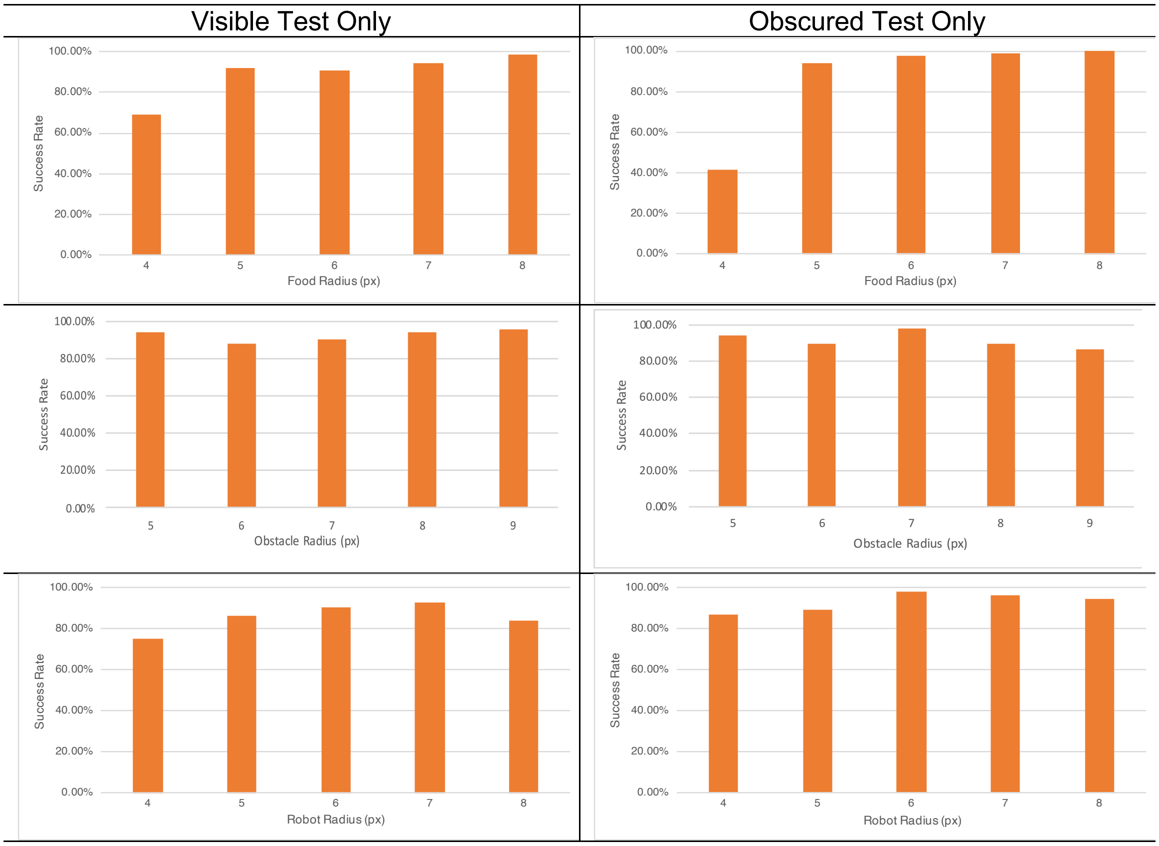

Fig. S9 Test success rate by varying the size of entities in the environment.

Visible Test Only

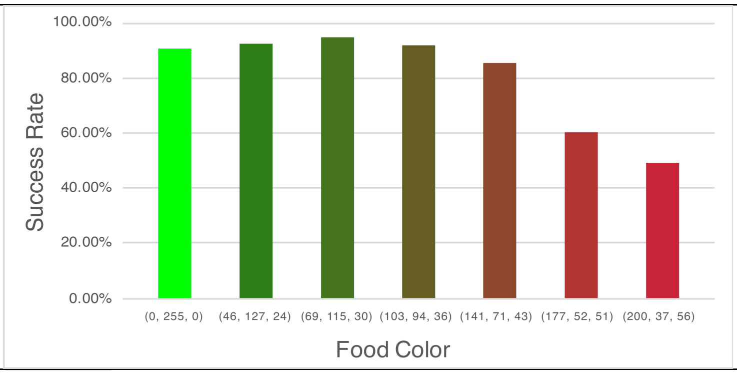

Obscured Test Only

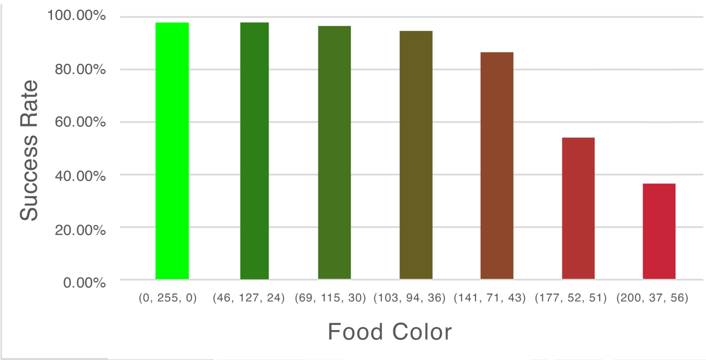

Visible Test Only

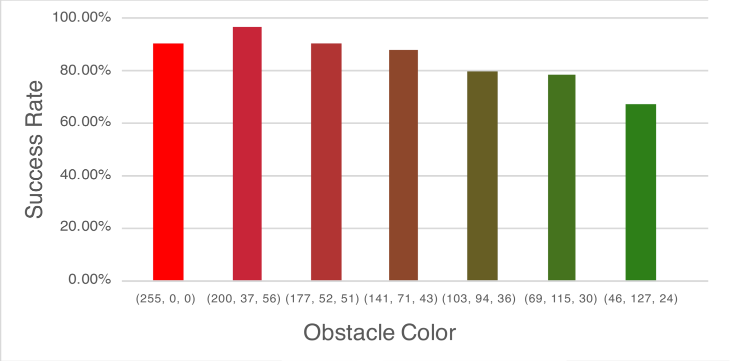

Obscured Test Only

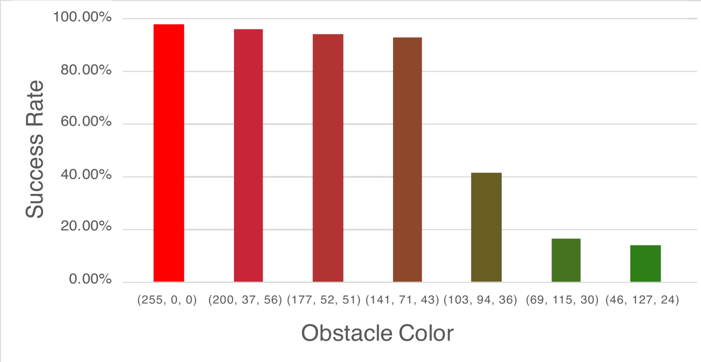

Fig. S10 Test success rate by varying the color of entities in the environment.

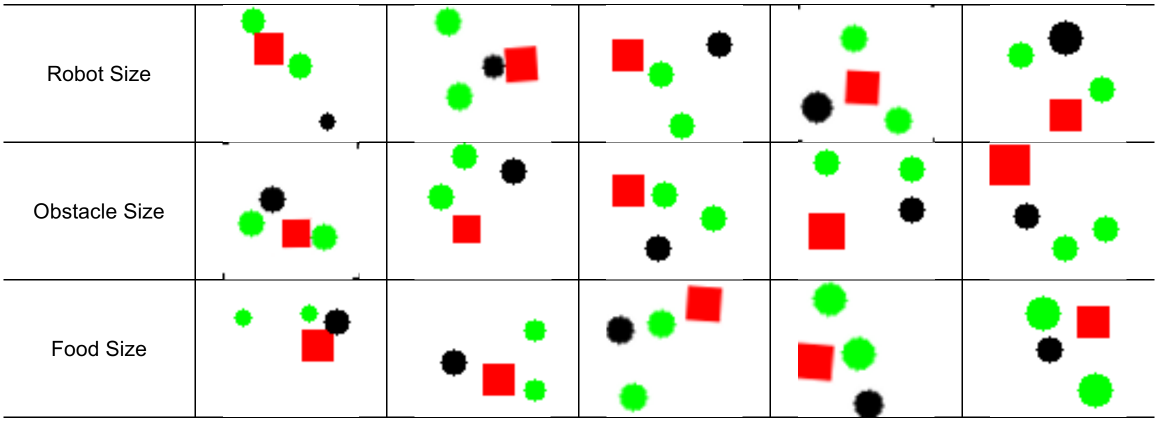

Fig. S11 Visualizations of all scenarios of size change.

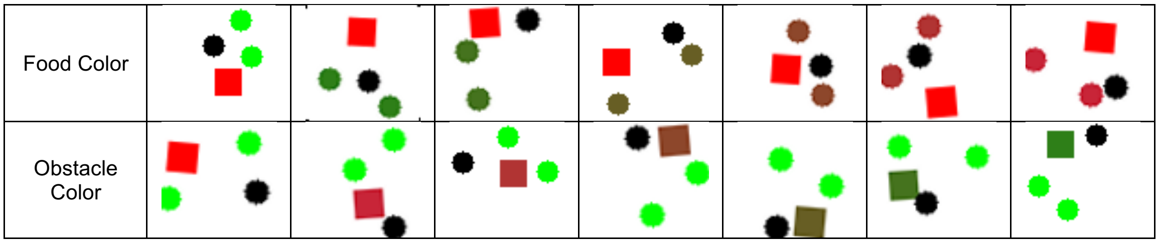

Fig. S12 Visualizations of all scenarios of color change.

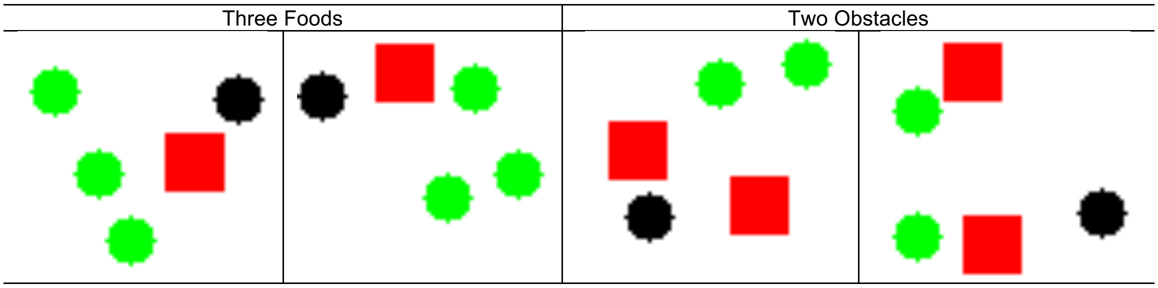

Fig. S13 Visualizations of all scenarios of number change.
